# Analysis of Matrix Metalloproteinase-8 Gene Polymorphisms and Their Association with Long-Term Survival, Clinical Phenotype, and Disease Risk in Patients with Coronary Artery Disease

**DOI:** 10.3390/ijms27156608

**Published:** 2026-07-24

**Authors:** Justyna Wrona, Anna Balcerzyk-Matić, Tomasz Nowak, Katarzyna Mizia-Stec, Artur Filipecki, Jolanta Krauze, Paweł Niemiec

**Affiliations:** 1 Department of Biochemistry and Medical Genetics, Faculty of Health Sciences in Katowice, Medical University of Silesia, Medykow Street 18, 40-752 Katowice, Poland; justyna.wrona@sum.edu.pl (J.W.); tnowak@sum.edu.pl (T.N.); pniemiec@sum.edu.pl (P.N.); 2 First Department of Cardiology, Faculty of Medical Sciences in Katowice, Medical University of Silesia, Ziolowa Street 47, 40-635 Katowice, Poland; kmiziastec@gmail.com (K.M.-S.); afilipecki@sum.edu.pl (A.F.); 3 1st Department of Cardiac Surgery/2nd Department of Cardiology, American Heart of Poland, S. A. Armii Krajowej Street 101, 43-316 Bielsko-Biala, Poland; jolakra@poczta.fm

**Keywords:** *MMP-8*, coronary artery disease, long-term survival, polymorphisms

## Abstract

Matrix metalloproteinase-8 (MMP-8), a neutrophil-derived collagenase responsible for the degradation of type I and III collagen, is involved in extracellular matrix remodeling, which may contribute to the destabilization of atherosclerotic plaques and thereby promote the development and progression of coronary artery disease (CAD). Single-nucleotide polymorphisms (SNPs) in the *MMP-8* gene may influence the expression and activity of this enzyme, potentially affecting disease risk, clinical manifestation, and long-term prognosis. The study group included 259 patients diagnosed with CAD and 239 control blood donors. Genotyping of *MMP-8* polymorphisms (rs1940475 and rs11225395) was performed using TaqMan PCR. *MMP-8* gene polymorphisms showed no association with CAD risk, disease severity, or patient survival at the 5- or 10-year follow-up. All studied polymorphisms are located within the same haplotype block, where commonly co-inherited alleles (T rs1940475 and A rs11225395) may exert opposing functional effects. In conclusion, these findings suggest no significant role of the analyzed *MMP-8* gene variants in CAD susceptibility or prognosis in the studied group.

## 1. Introduction

Matrix metalloproteinases (MMPs) constitute a family of zinc-dependent endopeptidases. These enzymes play an important role in maintaining the structure of blood vessel walls; however, they are also involved in the formation of atherosclerotic plaques and may contribute to their destabilization [1]. An important process regulated by MMPs, which contributes to plaque growth and consequently to the progression of atherosclerosis, is the remodeling of the extracellular matrix (ECM) structure of blood vessels [2]. MMPs may also influence the process of atherosclerotic plaque rupture by reducing the amount of collagen and other extracellular matrix proteins in the fibrous cap surrounding the plaque, thereby increasing its susceptibility to rupture [3].

One of the metalloproteinases that plays a significant role in the pathogenesis of atherosclerosis is neutrophil collagenase, encoded by the *MMP-8* (Matrix Metallopeptidase 8) gene [4]. Under physiological conditions, MMP-8 participates in the degradation of extracellular matrix components, particularly type I and type III collagen. It is secreted mainly by neutrophils in the form of a proenzyme in response to, among others, interleukin-1, TNF-α, and interleukin-8 [5,6].

Previous studies have shown elevated levels of MMP-8 in unstable carotid atherosclerotic plaques, which were associated with an increased risk of recurrent manifestation of atherosclerotic disease [7]. Serum MMP-8 concentrations also appear to influence the course of atherosclerosis-related diseases. According to Kormi I et al. [8], individuals with higher levels of this enzyme are at greater risk of future cardiovascular events, coronary artery disease, myocardial infarction, and death. Additionally, Fertin M et al. [9] found that baseline serum MMP-8 levels are a significant predictive factor for left ventricular remodeling and post-myocardial infarction prognosis, and may also help improve risk stratification. MMP-8 levels depend on many factors, including genetic variants of the *MMP-8* gene. Analyses of promoter polymorphisms of the *MMP-8* gene, rs11225395 (−799 C/T) and rs1320632 (−381 A/G), have shown that the −381G allele and the G−381T−799 haplotype were associated with higher mRNA expression in carotid atherosclerotic plaques, and thus may influence the risk of atherosclerosis in these arteries [10]. Another study demonstrated that the −799TT genotype (also reported as AA in alternative nomenclature) was significantly associated with increased serum MMP-8 levels [11], and experiments in melanoma cells showed that the A allele had approximately two-fold higher promoter activity than the G allele [12]. Another missense polymorphism, rs1940475 (c.259 T>C), is located in the pro-domain of *MMP-8*, and in vitro analysis by Laxton et al. [13] indicated that the MMP-8 zymogen produced by the T allele is less susceptible to activation than the zymogen produced by the C allele. Computational simulations have also shown that this polymorphism alters the structural stability of this domain, thereby reducing MMP-8 activation [14]. On the basis of previous evidence of functional relevance, including reported associations with gene expression, protein levels, and clinical phenotypes, we hypothesized that polymorphic variants of the *MMP-8* gene may be associated with the risk of coronary artery disease, its clinical course, and patient mortality. The selection criterion for the polymorphisms, apart from their functional relevance described previously, was a minor allele frequency (MAF) ≥ 0.20 to improve the potential statistical power of the analyses. The previously described rs1320632 polymorphism was not included in the study due to its low minor allele frequency (MAF = 0.09) in the European population. 

The main aim of this study was to analyze the association between two polymorphisms of the *MMP-8* gene (rs11225395 and rs1940475) and 5- and 10-year survival in patients with coronary artery disease (CAD), as well as the clinical phenotype of the disease. In addition, the influence of these polymorphisms on the risk of developing coronary artery disease was assessed.

## 2. Results

### 2.1. Analysis of the Associations Between Selected Polymorphisms of MMP-8 Gene and Coronary Artery Disease

The detailed characteristics of the studied groups, including risk factors for CAD, are presented in Table 1. These data were presented in our previous studies [15] and are provided for completeness. For all analyzed polymorphisms, the distribution of alleles and genotypes was consistent with the Hardy–Weinberg equilibrium (HWE) (Table S1). Genotype and allele frequencies did not differ significantly between the patients and control groups (Table 2).

**Figure 1:**
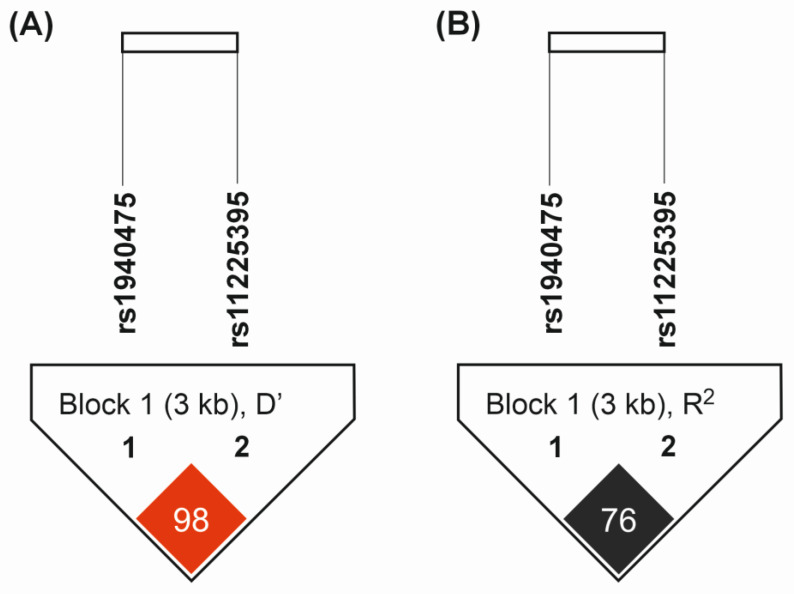
Haplotype analysis of *MMP-8* gene polymorphisms: (**A**) D’ value; (**B**) R^2^ value.

The analysis identified a haplotype block encompassing the two studied SNPs (rs1940475 and rs11225395), which were in strong linkage disequilibrium (Figure 1). No association was found between MMP-8 gene haplotypes and CAD.

The frequencies of haplotypes did not differ significantly between the patients and control groups (Table 3). Haplotypes with frequencies below 1% were not included in the analysis. 

### 2.2. Survival Analyses According to the Genotype of the Analyzed Polymorphisms

**Figure 2:**
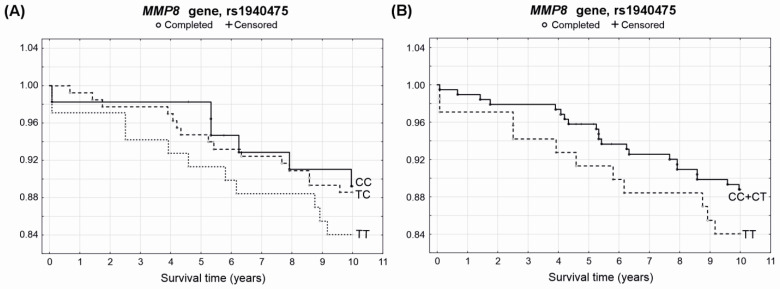
Kaplan–Meier curves in CAD patients stratified by rs1940475. (**A**) Additive model: χ^2^ = 2.902 (*p* = 0.234) at 5 years and χ^2^ = 1.160 (*p* = 0.560) at 10 years. (**B**) Recessive model: the log-rank statistic was −1.410 (*p* = 0.159) at 5 years and −1.036 (*p* = 0.300) at 10 years.

**Figure 3:**
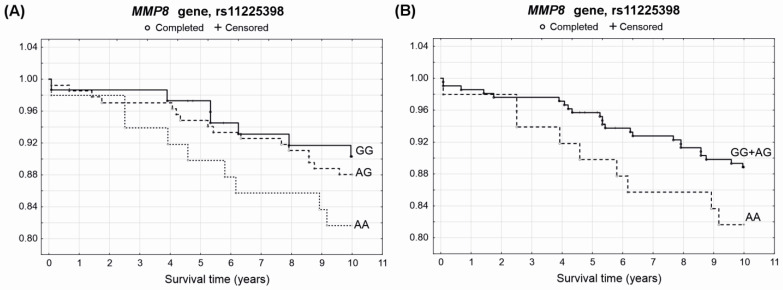
Kaplan–Meier curves in CAD patients stratified by rs11225395. (**A**) Additive model: χ^2^ = 3.166 (*p* = 0.205) at 5 years and χ^2^ = 2.224 (*p* = 0.329) at 10 years. (**B**) Recessive model: the log-rank statistic was −1.626 (*p* = 0.104) at 5 years and −1.392 (*p* = 0.164) at 10 years.

Among the 259 patients, the endpoint event occurred in 14 patients during the 5-year follow-up and in 32 patients during the 10-year follow-up. Kaplan–Meier survival analyses were performed for both additive and recessive models. No statistically significant differences in survival were observed among the genotypes of the three analyzed polymorphisms, either at the 5-year or 10-year follow-up (Figure 2 and Figure 3).

Both univariable and multivariable Cox proportional hazards models, adjusted for age, sex, smoking, diabetes, hypertension, lipid profile, and both analyzed polymorphisms, showed no significant associations with either 5-year or 10-year survival. No violations of the proportional hazards assumption were identified for the genetic variants (Table 4).

### 2.3. Association Between MMP-8 Polymorphisms and Clinical Features of CAD

In the CAD group, we investigated whether polymorphic variants of the *MMP-8* gene were associated with selected clinical features of patients, in particular with the clinical and angiographic parameters of coronary artery disease (Table 5). We performed univariable analysis for each polymorphism using additive and recessive/dominant models. No significant differences in genotype distribution were observed with respect to the analyzed parameters.

## 3. Discussion

The primary aim of this study was to analyze the impact of the investigated polymorphisms on patient survival during the 5- and 10-year follow-up periods, as well as the severity of the disease. We expected such associations, as previous studies have suggested that both analyzed polymorphisms influence gene expression and/or protein levels [10,11,12,13,14]. In turn, enzyme levels have been linked to coronary artery disease, myocardial infarction, recurrent cardiovascular events, or death [7,8,9]. The role of MMP-8 in the progression of atherosclerosis is most often linked to the degradation of type I collagen—the main component of the fibrous cap of atherosclerotic plaques—which promotes plaque destabilization and increases the risk of rupture and subsequent thrombus formation. Furthermore, this metalloproteinase modulates macrophage phenotype towards a pro-inflammatory state, influences plaque macrophage content, and induces the production of angiotensin II [16]. However, our analyses did not reveal any association between the studied polymorphisms and survival in either the 5- or 10-year follow-up. Although individuals carrying the AA (rs11225395) genotype, which has previously been associated with higher gene expression [11,12], showed slightly lower survival rates, these differences were not statistically significant. 

No association was found between the analyzed variants and either the clinical phenotype of CAD or the severity of atherosclerosis, expressed as the number of affected coronary arteries (one or multivessel disease) or the degree of stenosis. Such associations have previously been reported by Laxton et al. [13] for the rs1940475 polymorphism. They showed that the T allele had a lower frequency in patients who had >50% stenosis in two or three coronary arteries than in patients with >50% stenosis in one coronary artery. Moreover, in prospective studies, the authors observed that the T allele was associated with a protective effect against the progression of carotid artery atherosclerosis over a 10-year follow-up period. Discrepancies between our findings and those reported by Laxton et al. [13] may be explained by differences in study design, analyzed populations and sample size, as well as by variation in clinical profiles of the patients. Furthermore, although rs1940475 has demonstrated functional effects on MMP-8 activation in vitro and structural modeling suggests altered domain stability [13,14], these molecular effects may be attenuated in vivo by the complex regulation of matrix metalloproteinases. MMP-8 levels are influenced by several factors, including age, sex, smoking status, and obesity [17]. Additionally, the local inflammatory environment in atherosclerotic lesions, the cellular composition of the plaque, and the balance between MMP-8 and its endogenous inhibitors may further modulate the impact of genetic variation on MMP-8 activity [4]. However, the clinical significance of this polymorphism may also be modulated by linkage disequilibrium with a functionally opposing variant. Both analyzed polymorphisms are located within a single haplotype block, with the A allele (rs11225395) most frequently co-segregating with the T allele (rs1940475). rs11225395 was previously reported to influence promoter activity, with the A allele showing approximately two-fold higher promoter activity than the G allele [12]. The T allele of rs1940475 is a missense variant associated with reduced susceptibility of the zymogen to activation [14]. Thus, it appears that these two alleles exert opposing functional effects. Therefore, it can be hypothesized that the potential proatherogenic effect of rs1940475 may be partially counterbalanced by the influence of rs11225395. However, this hypothesis requires confirmation in studies assessing MMP-8 expression and serum MMP-8 concentrations and providing functional validation of the observed associations.

When selecting these two specific polymorphisms for this study, we considered the available experimental and clinical evidence, despite its limited amount, to provide more direct functional support than in silico predictions. However, we also analyzed the association between the studied polymorphisms and gene expression in silico, based on data from the GTEx Portal [18]. None of the investigated variants were associated with gene expression in the aorta, coronary arteries, whole blood or left ventricle. This may be explained by the previously described interplay between polymorphisms. However, it should also be noted that the GTEx project is based on samples from non-diseased tissues, whereas most of the studies on which we based our selection of polymorphisms were conducted in patients with cardiovascular diseases or in atherosclerotic plaques. In healthy tissues, MMP-8 is produced mainly by neutrophils, stored within specific granules, and released upon activation, where it contributes to connective tissue remodeling during acute inflammatory responses. However, in human atherosclerotic lesions, MMP-8 is also expressed by endothelial cells, vascular smooth muscle cells, and macrophages, and is markedly elevated in advanced, rupture-prone plaques compared with lesions characterized by a more stable morphology [19].

In our study, we also compared allele and genotype frequencies between patients with CAD and the control group. We found no statistically significant associations in either individual genotype comparisons or haplotype analyses. In light of our findings, a comparison of survival outcomes and atherosclerosis severity between individuals carrying the TG and CA haplotypes (rs1940475-rs11225395) would be of particular interest. However, such analyses could not be performed in the present study due to the very low frequency of these haplotypes in the study population.

The main limitations of our study are the relatively small patient cohort (*N* = 259) and the limited number of endpoints. During the 5-year follow-up there were 14 events, while during the 10-year follow-up there were 32 events, which affected the statistical power of the analyses. Furthermore, the small number of patients with specific haplotypes prevented more detailed analyses. Given the single-center design and the relatively homogeneous study population, our findings should be interpreted with caution when generalized to populations with different demographic and genetic characteristics. Due to the retrospective design, no biological material was available to assess the relationship between genotype and mRNA expression or MMP-8 protein levels. Therefore, the biological interpretation relies solely on the functional effects of these polymorphisms reported in previous studies. Additionally, some limitations may arise from the selection of the control group, which consisted of blood donors without symptoms or a history of CAD. As no diagnostic procedures were performed, subclinical atherosclerosis may have led to the misclassification of some controls. However, this limitation affects only the association analysis of *MMP-8* variants with CAD risk, as survival analyses and genotype–phenotype associations were conducted within the CAD group only. 

## 4. Materials and Methods

### 4.1. Characteristics of the Study Group

The study cohort consisted of 259 patients with angiographically documented premature coronary artery disease (CAD), comprising 79 women and 180 men. The median age of the patient group at study enrollment was 46.00 ± 5.00 years (±QD). The control group consisted of 239 healthy blood donors (67 women and 172 men) with a median age at enrollment of 44.00 ± 4.00 years, who had no clinical manifestations of CAD, no previous myocardial infarction (MI), and no family history of cardiovascular disease. Premature CAD was defined as the presence of at least 50% stenosis in one or more major coronary arteries confirmed by coronary angiography performed according to the Judkins technique. Individuals with coagulopathies, diagnosed cardiomyopathies, collagen vascular diseases, or acute intoxications (including carbon monoxide or amphetamine poisoning) were excluded from the study. The baseline characteristics of the study groups have been described previously [15] and are presented in Table 1. The biological material used in the study was collected between 2001 and 2013 and stored at the Department of Biochemistry and Medical Genetics. The study protocol adhered to the ethical principles outlined in the Declaration of Helsinki and was approved by the Bioethics Committee of the Medical University of Silesia in Katowice (KNW/0022/KB1/17/I/11, NN-013-107/I/00). Written informed consent was obtained from all participants prior to their enrollment in the study.

### 4.2. Genetic Analysis

Genomic DNA was extracted from peripheral blood leukocytes using the MasterPure™ DNA Purification Kit (Epicentre Technologies, Madison, WI, USA). Selected polymorphisms within the *MMP8* gene were genotyped using TaqMan^®^ SNP Genotyping Assays (Thermo Fisher Scientific, Waltham, MA, USA). Real-time PCR amplification and allelic discrimination were carried out using the LightCycler 480 Real-Time PCR System (F. Hoffmann-La Roche AG, Basel, Switzerland). The analyzed *MMP8* polymorphic variants (rs1940475 and rs11225395) were selected based on previously reported associations with *MMP-8* expression, protein level, or clinical phenotypes, as well as according to the minor allele frequency (MAF ≥ 0.20) in European populations. The summary of the analyzed polymorphisms is presented in Table 6.

### 4.3. Statistical Analysis

Statistical analyses were conducted using STATISTICA 13.0 software (TIBCO Soft-ware Inc., Palo Alto, CA, USA). Allele frequencies were calculated from genotype distributions, and compliance with Hardy–Weinberg equilibrium was assessed using the χ^2^ test. Comparisons of all qualitative variables were performed using Pearson’s chi-square test in univariable analysis. Yates’ correction was applied for subgroups with fewer than ten subjects, whereas Fisher’s exact test was used when subgroup sizes were below five. Results were reported as odds ratios (ORs) with 95% confidence intervals (CIs) and corresponding *p* values. A *p* value below 0.05 was considered statistically significant. Cases with missing data were excluded from the relevant analyses (this applies mainly to the clinical phenotype of CAD). The Bonferroni–Hochberg correction was applied to account for multiple testing [20]. All *p*-values corresponding to a given research hypothesis were considered simultaneously, for example, when assessing whether a specific polymorphism (across all genetic models) was associated with cardiovascular disease status or influenced patient survival. Haplotype analysis based on the examined polymorphisms was performed using the Haploview 4.2 software. Linkage disequilibrium was calculated based on the algorithm by Gabriel et al. [21]. This algorithm uses D’ and R^2^ values, and a haplotype block creates a contiguous set of polymorphisms for which recombination is absent or very rare. Linked polymorphisms are considered those with a D’ value between 0.7 and 1. However, values below 0.9 may indicate recombination, so the R^2^ value, which should be greater than 0.7 (70%), is also used.

### 4.4. Survival Analysis

Cardiovascular mortality, defined according to the ICD-10 classification (Table S2), was adopted as the study endpoint. Information regarding causes and dates of death was obtained from the Katowice City Hall and the Central Statistical Office of Poland. Survival analyses were performed using STATISTICA 13.0 software. Kaplan–Meier curves were generated to estimate survival probabilities, and intergroup differences were evaluated using the log-rank test. The association between *MMP8* genetic variants and mortality risk was evaluated using Cox proportional hazards regression in univariable and multivariable models. The multivariable models were adjusted for age, sex, smoking, diabetes, hypertension, lipid profile, and both analyzed polymorphisms. Results were expressed as hazard ratios (HRs) with corresponding 95% CIs. The proportional hazards assumption was verified for all analyses. Statistical significance was defined as *p* < 0.05. 

## 5. Conclusions

In conclusion, the present study did not provide evidence of an association between the analyzed *MMP-8* gene polymorphisms and the risk of coronary artery disease, disease severity, or patient survival. However, given the limited statistical power of the study, a potential association cannot be completely excluded. Therefore, these findings should be interpreted with caution and require validation in larger, multicenter cohorts. Both studied polymorphisms are located within the same haplotype block, where commonly co-inherited alleles (T rs1940475 and A rs11225395) may exert opposing functional effects. 

## Figures and Tables

**Table 1:** Risk factors of CAD in patients and controls (blood donors).

Characteristics	CAD Patients		Controls				*p*
	*n* *	*n*	%	*n* *	*n*	%	
*N*		259			239		
Male sex	259	180	69.50	239	172	71.97	0.545
Hypertension	255	205	80.39	105	9	8.57	0.000
DM	256	22	8.59	239	0	0.00	0.000
Cigarette smoking	219	143	65.30	231	66	28.57	0.000
Overweight/obesity (BMI ≥ 25)	238	162	68.07	200	116	58.00	0.029
Obesity (BMI ≥ 30)	238	50	21.01	200	43	21.50	0.900
	*n* *	Median	±QD	*n* *	Median	±QD	
Age [years]	251	46.0	5.00	229	44.00	4.00	0.071
BMI [kg/m^2^]	238	26.70	2.60	200	25.40	2.60	0.016
TC [mmol/L]	246	5.80	0.92	200	5.21	0.74	0.000
HDL [mmol/L]	242	1.06	0.16	199	1.22	0.25	0.000
LDL [mmol/L]	243	4.25	0.87	199	3.62	0.82	0.000
TG [mmol/L]	245	1.81	0.53	199	1.40	0.47	0.000

Legend: BMI, body mass index; DM, diabetes mellitus; TC, total cholesterol; HDL, high-density lipoprotein cholesterol; LDL, low-density lipoprotein cholesterol; TG, triglycerides; *n* *—number of subjects in respective analyses.

**Table 2:** Comparison of genotype and allele frequencies of the *MMP-8* gene in CAD patients and controls.

Genotype/ Allele	CAD Patients ^1^ *n* (%)	Controls ^2^ *n* (%)	Inheritance Model	OR (95% CI)	*p*
rs1940475					
CC	57 (22.01)	65 (27.20)	Dominant, vs. CT+TT	0.76 (0.50–1.38)	0.179
TC	133 (51.35)	110 (46.02)	Additive, vs. CC	1.38 (0.89–2.13)	0.148
TT	69 (26.64)	64 (26.78)	Recessive, vs. CC+CT	0.99 (0.67–1.48)	0.972
C	247 (47.68)	240 (50.21)	-	0.90 (0.70–1.16)	0.426
T	271 (52.32)	238 (49.79)	-	1.11 (0.86–1.42)	0.426
rs11225395					
GG	74 (28.68)	79 (33.19)	Dominant, vs. AG + AA	0.81 (0.55–1.19)	0.277
AG	135 (52.33)	105 (44.12)	Additive, vs. GG	1.37 (0.91–2.06)	0.127
AA	49 (15758.99)	54 (22.69)	Recessive, vs. GG + AG	0.80 (0.52–1.23)	0.311
G	283 (54.84)	263 (55.25)	-	0.98 (0.77–1.26)	0.898
A	233 (45.16)	213 (44.75)	-	1.02 (0.79–1.31)	0.898

Legend: OR, Odds Ratio; CAD, Coronary Artery Disease (patient group). ^1^ Data for 259 participants, except for rs11225395 (n = 258 due to missing genotype data). ^2^ Data for 239 participants, except for rs11225395 (n = 238 due to missing genotype data).

**Table 3:** Comparison of haplotype frequencies between CAD patients and controls.

Haplotypes	Frequency		OR (95% CI)	*p*
rs1940475/rs11225395	CAD Patients *n* (%)	Controls *n* (%)		
CC/GG	56 (21.71)	64 (26.89)	0.75 (0.50–1.14)	0.178
CC/AG	1 (0.39)	1 (0.42)	-	-
CC/AA	0 (0.00)	0 (0.00)	-	-
TC/GG	16 (6.20)	15 (6.30)	0.98 (0.47–2.03)	0.963
TC/AG	116 (44.96)	93 (39.08)	1.27 (0.89–1.82)	0.185
TC/AA	0 (0.00)	1 (0.42)	-	-
TT/GG	2 (0.78)	0 (0.00)	-	-
TT/AG	18 (6.98)	11 (4.62)	1.55 (0.72–3.35)	0.264
TT/AA	49 (18.99)	53 (22.27)	0.82 (0.53–1.27)	0.367

**Table 4:** Results of the Cox proportional hazards model with verification of the proportional hazards assumption in the overall study group.

Inheritance Model	Follow-Up	*p*	HR (95%CI)	*p* for PH
rs1940475				
Additive	5 years	0.093	0.50 (0.22–1.12)	0.712
	10 years	0.348	0.79 (0.47–1.30)	0.460
Recessive	5 years	0.167	0.47 (0.16–1.37)	0.780
	10 years	0.301	0.68 (0.33–1.41)	0.534
rs11225395				
Additive	5 years	0.087	1.98 (0.91–4.31)	0.565
	10 years	0.168	1.43 (0.86–2.37)	0.645
Recessive	5 years	0.114	2.42 (0.81–7.21)	0.618
	10 years	0.165	1.73 (0.80–3.73)	0.686

Legend: HR, Hazard Ratio; PH, Proportional Hazards.

**Table 5:** Variants of the *MMP-8* gene polymorphisms and clinical phenotype of CAD.

Characteristic		*n* *	Genotypes, *n* (%)			*p* Value for Models	
						Additive	Recessive/Dominant
rs1940475			CC	CT	TT		TT vs. CC/CT
MI	Yes	258	37 (21.26)	88 (50.57)	49 (28.16)	0.740	0.459
	No		20 (23.81)	44 (52.38)	20 (23.81)		
ACS	Yes	214	42 (22.34)	94 (50.00)	52 (27.66)	0.883	0.797
	No		6 (23.08)	14 (53.85)	6 (23.08)		
UA	Yes	152	9 (26.47)	16 (47.06)	9 (26.47)	0.800	0.885
	No		25 (21.19)	61 (51.69)	32 (27.12)		
Multivessel stenosis	Yes	245	15 (25.86)	28 (48.28)	15 (25.86)	0.723	0.832
	No		39 (20.86)	97 (51.87)	51 (27.27)		
Critical stenosis	Yes	234	32 (20.38)	80 (50.96)	45 (28.66)	0.496	0.666
(≥90%)	No		21 (27.27)	36 (46.75)	20 (25.97)		
rs11225395			GG	AG	AA		AA vs. GG/AG
MI	Yes	257	51 (29.31)	87 (50.00)	36 (20.69)	0.530	0.337
	No		23 (27.71)	47 (56.63)	13 (15.66)		
ACS	Yes	213	57 (30.32)	93 (49.47)	38 (20.21)	0.523	0.426
	No		7 (28.00)	15 (60.00)	3 (12.00)		
UA	Yes	151	14 (41.18)	14 (41.18)	6 (17.65)	0.205	0.988
	No		30 (25.64)	64 (54.70)	23 (19.66)		
Multivessel stenosis	Yes	244	19 (32.76)	29 (50.00)	10 (17.24)	0.729	0.719
	No		51 (27.42)	99 (53.23)	36 (19.35)		
Critical stenosis (≥90%)	Yes	233	42 (26.75)	84 (53.50)	31 (19.75)	0.465	0.999
	No		26 (34.21)	35 (46.05)	15 (19.74)		

Legend: MI, Myocardial Infarction; ACS, Acute Coronary Syndrome; UA, Unstable Angina; *, number of CAD patients in respective comparisons.

**Table 6:** Summary of *MMP-8* single-nucleotide polymorphisms analyzed in the study.

SNP ID	Chromosomal Position	Genome Build	Nucleotide Change
rs1940475	chr11:102722517	GRCh38.p14	c.259 T>C
rs11225395	chr11:102725749	GRCh38.p14	−799 A>G

## Data Availability

The data supporting the findings of this study are available within the article. Individual-level data are not publicly available due to ethical and privacy restrictions but may be obtained from the corresponding author upon reasonable request and subject to approval by the relevant ethics committee.

## Supplementary Materials

The following supporting information can be downloaded at https://www.mdpi.com/article/10.3390/ijms27156608/s1.

## Abbreviations

The following abbreviations are used in this manuscript:

MMPs	Matrix metalloproteinases
SNPs	Single-nucleotide polymorphisms
ECM	Extracellular matrix
MMP-8	Matrix Metallopeptidase 8
CAD	Coronary artery disease
ACS	Acute coronary syndrome
AMI	Acute myocardial infarction
OR	Odds ratio
CI	Confidence interval
HR	Hazard ratio
PH	Proportional hazards
MI	Myocardial infarction
MAF	Minor allele frequency
HWE	Hardy–Weinberg equilibrium
LD	Linkage disequilibrium
