# Supplementary material for: Analysis of Matrix Metalloproteinase-8 Gene Polymorphisms and Their Association with Long-Term Survival, Clinical Phenotype, and Disease Risk in Patients with Coronary Artery Disease
Source: Int J Mol Sci. 2026 Jul 24;27(15):6608. doi: 10.3390/ijms27156608 (PMC13466682; doi:10.3390/ijms27156608)
Supplement: Supplementary file 1 [file ijms-27-06608-s001.zip › ijms-4383900-supplementary.pdf]

Table S1. Hardy–Weinberg equilibrium analysis of the studied polymorphisms.

SNP	Study group	Genotype distribution	HWE *p*-value
rs1940475	Controls	CC=65, TC=110, TT=64	0.219
	CAD Patients	CC=57, TC=133, TT=69	0.638
rs11225395	Controls	GG=79, AG=105, GG=54	0.096
	CAD Patients	GG=74, AG=135, AA=49	0.365

Table S2. Cardiovascular causes of death in 5-year and 10-year follow-up.

ICD-10 code	Cause of death	5-year follow-up		10-year follow-up	
		n	%	n	%
I20.8	Ischaemic heart diseases. Other forms of angina pectoris.	0	0,00	1	3,13
I21.0	Ischaemic heart diseases. Acute transmural myocardial infarction of anterior wall.	0	0,00	1	3,13
I21.9	Ischaemic heart diseases. Acute myocardial infarction, unspecified.	6	42,86	9	28,13
I22.9	Ischaemic heart diseases. Subsequent myocardial infarction of unspecified site.	2	14,29	3	9,38
I24.9	Ischaemic heart diseases. Acute ischaemic heart disease, unspecified.	0	0,00	1	3,13
I25.0	Ischaemic heart diseases. Atherosclerotic cardiovascular disease, so described.	1	7,14	1	3,13
I25.1	Ischaemic heart diseases. Atherosclerotic heart disease.	0	0,00	1	3,13
I25.5	Ischaemic heart diseases. Ischaemic cardiomyopathy.	0	0,00	2	6,25
I25.9	Ischaemic heart diseases. Chronic ischaemic heart disease, unspecified.	2	14,29	6	18,75
I46.9	Other forms of heart disease. Cardiac arrest, unspecified.	0	0,00	1	3,13
I50.1	Other forms of heart disease. Left ventricular failure.	1	7,14	1	3,13
I61.6	Cerebrovascular diseases. Intracerebral haemorrhage, multiple localized.	0	0,00	1	3,13
I63.5	Cerebrovascular diseases. Cerebral infarction due to unspecified occlusion or stenosis of cerebral arteries.	1	7,14	1	3,13
I63.9	Cerebrovascular diseases. Cerebral infarction, unspecified.	1	7,14	1	3,13
I70.2	Diseases of arteries, arterioles and capillaries. Atherosclerosis of arteries of extremities.	0	0,00	1	3,13
I70.9	Diseases of arteries, arterioles and capillaries. Generalized and unspecified atherosclerosis.	0	0,00	1	3,13
Σ		14	100,00	32	100,00
